# Adductomics of Newborn Dried Blood Spots Detects Constituents of Maternal Smoking During Pregnancy and Associated Oxidative Stress Exposure

**DOI:** 10.3390/antiox15040411

**Published:** 2026-03-25

**Authors:** Dean Madera, Yeunook Bae, Fariba Tayyari, Aishwarya Jala, Rohit Varma, William E. Funk, Joseph L. Wiemels, Xuejuan Jiang

**Affiliations:** 1 Department of Population and Public Health Sciences, University of Southern California Keck School of Medicine, Los Angeles, CA 90033, USA; dean.s.madera@gmail.com (D.M.); wiemels@usc.edu (J.L.W.); 2 School of Osteopathic Medicine in Arizona, A.T. Still University, Mesa, AZ 85209, USA; 3 Department of Health Sciences, Illinois State University, Normal, IL 61790, USA; ybae2@ilstu.edu; 4 Department of Preventive Medicine, Feinberg School of Medicine, Northwestern University, Chicago, IL 60611, USA; ftayyari@northwestern.edu (F.T.); aishwarya.jala@northwestern.edu (A.J.); w-funk@northwestern.edu (W.E.F.); 5 Southern California Eye Institute, Los Angeles, CA 90027, USA; rvarma@sceyes.org; 6 Department of Ophthalmology, University of Southern California Keck School of Medicine, Los Angeles, CA 90033, USA

**Keywords:** human serum albumin (HSA) adduct, cysteine 34 (Cys^34^), smoking, cotinine, prenatal, acrylonitrile, cyanide

## Abstract

Maternal Smoking During Pregnancy (MSDP) remains a major source of fetal toxicant exposure. We applied adductomics to profile reactive adducts at the human serum albumin cysteine-34 (HSA-Cys^34^) locus, which integrates longer-term exposures. HSA-Cys^34^ adducts formed by acrylonitrile and ethylene oxide, two tobacco-related toxicants previously linked to smoking in adults, were quantified and compared with cotinine and MSDP status. Their relationships with other reactive adducts were also examined. Neonatal dried blood spots (DBS) from 110 children were analyzed. Cotinine and 55 Cys^34^ adducts were measured by Liquid Chromatography–Tandem Mass Spectrometry (LC-MS/MS). Associations were evaluated using linear regression, chi-square tests, and principal component analysis. Eighteen adducts differed significantly by MSDP status after Bonferroni correction (*p* ≤ 9.1 × 10^−4^). S-acrylonitrile was markedly elevated in exposed newborns, including those whose mothers reported smoking cessation after early pregnancy (*p* < 0.001). S-acrylonitrile correlated with 31 adducts related to oxidative stress and thiol metabolism, whereas cotinine correlated with eight. S-ethylene oxide, though detectable in DBS, showed no consistent association with MSDP. Adductomics analysis of newborn DBS sensitively captures molecular signatures of prenatal tobacco exposure and related oxidative stress. Acrylonitrile adducts appear to better reflect cumulative MSDP exposure than cotinine, highlighting the utility of adductomics for improved exposure assessment and mechanistic insight.

## 1. Introduction

Intrauterine exposure to adverse conditions, such as malnutrition or environmental toxins, can significantly impair fetal development and lead to long-term health consequences for children. For example, maternal smoking during pregnancy (MSDP) is consistently identified as a risk factor for several pediatric conditions, including low birthweight and vision disorders such as strabismus, refractive errors, and retinopathy [1,2]. However, many epidemiological and public health studies have relied on self-reported data and maternal biomarker measurements that may not accurately reflect fetal exposure levels. For instance, self-reported smoking by pregnant women often underestimates the true extent of MSDP [3]. Cotinine, a metabolite of nicotine, has been studied as the primary biomarker for smoking exposure [4], but its short half-life of approximately 24 h limits its utility in capturing cumulative smoking exposure [5].

Recent advances in high-resolution and high-throughput technologies have enabled the development of methodology to systematically measure protein adducts to human serum albumin (HSA) in biospecimens [6]. With a half-life of around 20 days, HSA allows a longer window for the measurement of protein adducts during chronic exposures [7]. Most studies on HSA adducts have focused on adducts to the Cys^34^ loci, the primary scavenger of reactive electrophiles in circulating blood. This approach has contributed to disentangling the complex exposure profiles associated with smoking and air pollution [8]. For example, Grigoryan et al. characterized 43 Cys^34^ adducts using archived plasma from healthy volunteers aged 18–35 years. They reported that adducts of the ethylene oxide and acrylonitrile—two suspected human carcinogens found in cigarette smoking, marijuana, and e-cigarettes—were significantly higher in smokers, making them potential biomarkers for distinguishing smokers from nonsmokers [8]. Conversely, S-Sulfinic acid and S-Cys (a biomarker of antioxidant defense) adducts were lower in smokers compared to nonsmokers.

A few studies have also applied adductomics to newborn dried blood spots (DBS), which have emerged as a cost-effective resource for biomarkers of intrauterine exposure in large-scale public health and epidemiological research. Despite the limited blood volume from DBS, Funk et al. [9] reliably detected a targeted panel of 15 adducts in newborn DBS. They found that exposure to air pollution in the last 30 days of pregnancy was associated with lower levels of S-sulfinic acid and S-γ-GluCys and a higher level of S-crotonaldehyde, all indicative of increased oxidative stress. Yano et al. [10] measured 26 untargeted Cys^34^ adducts in 46 newborn DBS and found that Cys^34^ adducts of cyanide were significantly higher among newborns of smoking mothers compared to those of nonsmoking mothers. However, contrary to observations from the study of plasma samples from young healthy adults [8], this study did not detect Cys^34^ adducts of ethylene oxide and acrylonitrile in newborn DBS from smoking mothers. It is suspected that these mothers might have ceased smoking during the third trimester or before the last month of pregnancy. Additionally, the study found that an adduct with m/z of 830.43, suspected to represent addition of acrolein to Cys^34^, was lower in newborns of smoking mothers. This finding contradicts prior reports of higher levels of acrolein metabolites in the urine of users of conventional cigarette and e-cigarette-only users [11,12]. Further investigations are needed to validate these observations and explore the conflicting findings.

In this study, we present new findings from a high-throughput, targeted triple-quadrupole (QqQ) assay with enhanced sensitivity and specificity for detecting low-abundance HSA adducts. By utilizing a targeted approach rather than previous untargeted methods, we were able to quantify 55 specific Cys^34^ adducts in newborn DBS from 110 children. This same panel of adducts has also been successfully detected in cord blood [13]. We evaluated their associations with detailed parent-reported MSDP data and cotinine levels quantified in DBS samples. DBS has several practical advantages: low cost, minimal invasiveness, easy collection, and high storage efficiency. Because DBS collection is standard practice among newborns in the U.S., it is a readily available matrix for large-scale biomonitoring and research [14]. Despite challenges such as variable blood volume, higher sample complexity, and biomarker stability issues, Liquid Chromatography–Tandem Mass Spectrometry (LC-MS/MS)-based assays for DBS have been shown to be highly specific and multiplexable. Our investigation into highly sensitive and high-throughput adductomics offers new insights into the impact of MSDP.

## 2. Materials and Methods

### 2.1. Study Population and Samples

The study protocol was reviewed and approved by the Institutional Review Board (IRB)/Ethics Committee of the Los Angeles County/University of Southern California Medical Center and the California Health and Human Services Agency. The study complied with the Health Insurance Portability and Accountability Act (HIPAA) regulations. A parent or guardian of each study participant gave written informed consent for participating in the Multiethnic Pediatric Eye Disease Study (MEPEDS).

Subjects were selected from the MEPEDS, a population-based study of vision disorders in children between the ages of 6–72 months from Los Angeles County, CA, USA. The study obtained informed consent from the parents or guardians of each eligible participant. A study interviewer administered a questionnaire to each child’s parent at the time of the clinical examination or by telephone if the child was accompanied to the exam by someone other than a parent. The questionnaire included sociodemographic, maternal factors during pregnancy, and children’s factors during early childhood questions. Specifically, questions about maternal smoking during pregnancy were only asked if the biological mother of the child was answering the questionnaire. Mothers were asked whether they smoked at any time during the pregnancy of the participating child, and if yes, during which months of the pregnancy and how many cigarettes per day on average. Based on the information, status of maternal smoking during pregnancy was coded as nonsmokers, smoking in the 1st or 2nd trimester pregnancy only, and smoking persisting into the 3rd trimester.

Neonatal blood specimens (as dried blood spots) from MEPEDS children were obtained from California’s Biobank Program (SIS request # 1422) with a waiver of consent from the Committee for the Protection of Human Subjects of the State of California. The program archives biospecimens from the California Genetic Disease Screening Program (GDSP). GDSP screens newborns in California for genetic and congenital disorders. Newborn blood was stored as DBS collected on cards known as Guthrie cards. Newborn DBS were retrieved from 1456 children who participated in the MEPEDS, including equal numbers with common ocular diseases (e.g., hyperopia, astigmatism, strabismus) and with normal vision, individually matched on age, sex, and race/ethnicity. Out of these children, 46 had parent-reported maternal smoking throughout all three trimesters of pregnancy, 44 had maternal smoking during the first and/or second trimester only. DBS from these children were selected for the adductomics analyses. Additionally, we randomly selected DBS from 20 children without parent-reported maternal smoking during pregnancy for comparison.

### 2.2. HSA Isolation from Newborn DBS

HSA was isolated from DBS samples using the methods described previously [9]. Briefly, 3.2 mm DBS punches were extracted in a 45% methanol solution to precipitate hemoglobin and other interfering proteins. Samples were then agitated at room temperature for 30 min and centrifuged for 15 min at 4 °C to remove precipitates and cellulose fibers. The supernatant was diluted with digestion buffer, filtered, and then trypsin digested. Protein digestion was performed using a Barozyme HT48 (Pressure Biosciences™, Canton, MA, USA). After digestion, 10% formic acid was added to denature the trypsin and samples were centrifuged for 15 min at 14,000× *g*. A 100 μL aliquot of the digest was used for targeted adductomics analyses.

### 2.3. Targeted Adductomics

Adduct quantification and data processing have been described in detail previously [9,13]. A panel of 55 adducts (Table S1) was chosen based on previous associations with tobacco smoking and oxidative stress-related processes [9,13]. Our panel included adducts of toxicants found in tobacco smoke (acrylonitrile and ethylene oxide), direct oxidation products (e.g., S-sulfinic acid and S-sulfonic acid) that are directly formed through reactions with reactive oxygen species (ROS), reactive aldehydes (e.g., acrolein, crotonaldehyde) generated when ROS interact with polyunsaturated fatty acids, and small thiol compounds (e.g., Cys, GSH, γ-GluCys, and CysGly) that can serve as biomarkers of antioxidant defense.

LC–MS/MS analyses were performed on an Agilent 1260 Infinity HPLC system coupled to an Agilent 6490 triple-quadrupole mass spectrometer, following the method described by Funk et al. (2021) [9]. Chromatographic separations were achieved on Agilent Poroshell 120 column (3 × 50 mm, 2.7 μm) with a 10-min gradient at a flow rate of 0.7 mL/min. The mobile phase consisted of solvent A (0.1% *v*/*v* formic acid in water) and solvent B (100% acetonitrile). The column was maintained at 37 °C and the injection volume was 10 µL. The mass spectrometer operated with an iFunnel electrospray ionization source and JetStream technology using dynamic Multiple Reaction Monitoring mode (dMRM). dMRM transitions, collision energies, and all other mass spectrometric parameters were applied as described by Funk et al. [9]. Adduct levels were derived based on the summed peak areas of three transitions per adduct species and normalized to the combined peak areas of the three transition peaks of the housekeeping peptide, which is an HSA peptide adjacent to the T3 peptide where the adducts are formed.

The limit of quantification for each adduct was calculated as 10 times the standard deviation of ion intensities from multiple blank samples. Blank samples were analyzed within each sample batch. Levels below the limit of detection (3 times the noise) were retained for statistical analyses. These values represent meaningful biological signals rather than random noise, as they are supported by consistent detection across three specific ion transitions.

Adduct stability has been rigorously evaluated, and adductomics has been successfully applied to DBS samples stored for up to 20 years. During analytical runs, following extraction and preparation, adducts remained highly stable for at least 24 h, corresponding to the maximum runtime required for a batch of 30 samples. Nonetheless, to account for variability related to storage conditions and batch effects, pooled quality control samples were included and monitored in each analytical batch.

### 2.4. Cotinine Extraction from Dried Blood Spots

DBS samples were processed following the extraction and LC–MS/MS conditions described in Ladror et al. (2017) [15]. Briefly, 3.2 mm DBS punches were extracted in 100 µL of methanol with 25 ng/mL of d3-cotinine added as an internal standard. Extraction was performed using a Barozyme HT48 cycled between atmospheric pressure and 20 kpsi. Chromatographic separation was carried out on an Agilent Poroshell 120 column (EC-C^18^, 3 × 50 mm, 2.7 µm, Agilent Technologies, Santa Clara, CA, USA) using the mobile-phase gradient reported previously, and cotinine was quantified on an Agilent 6490 triple-quadrupole mass spectrometer coupled to an Agilent 1260 Infinity HPLC (Agilent Technologies, Santa Clara, CA, USA). Mass-spectrometric parameters, including MRM transitions and collision energies, followed those described in Ladror et al. [15]. This method has been shown to reliably classify smoking status, demonstrating 100% sensitivity and 94% specificity for distinguishing smokers from nonsmokers in adult DBS samples.

### 2.5. Statistical Analysis

Quantitation of adducts is achieved by calculating the Peak Area Ratio (PAR), which is the ratio of the adduct peak area to that of a housekeeping (HK) peptide. The PAR provides a reliable measure of adduct concentration across a wide dynamic range [8]. Data on adducts were log-transformed before analyses. Parent-reported MSDP was analyzed based on (1) the timing of smoking—nonsmoker, smoked during 1st/2nd trimester only, and smoked through three trimesters—and (2) based on the dose of smoking—no maternal smoking, mother smoked less than 5 cigarettes/day during pregnancy, and mother smoked greater than 5 cigarettes/day.

Differences in adducts between two groups (e.g., with and without MSDP, MSDP in 1st/2nd vs. 3rd trimester) were compared using *t*-tests. Volcano plots were generated based on *t*-test results. We also applied Wilcoxon rank sum tests for between-group differences, and results were similar to those from generalized linear regression. Generalized linear regression tests were used to evaluate the individual associations of Cys^34^ adducts with MSDP, cotinine level in DBS, and gestational age of the children.

To reduce the dimensionality of the Cys^34^ adduct data (excluding acrylonitrile, cotinine, and ethylene oxide), we applied principal component (PC) analysis to identify patterns in the correlations between adducts. We extracted linear composites (i.e., factors) of the adducts that explain the largest proportion of total variance in the adducts. Factors with Eigenvalues greater than or equal to 2.0 were further analyzed for their associations with MSDP, cotinine, and acrylonitrile.

All statistical analyses were conducted using SAS 9.4 (SAS Institute Inc., Cary, NC, USA). Bonferroni correction was applied for tests across all HSA adducts. Statistical significance was defined as *p*-values less than 0.05 for all other tests.

## 3. Results

The study cohort consisted of 110 children (Table S2). Sixty-two were male, approximately 85% were African American, and 82% were born at full term (defined as gestational age of 37 weeks or longer).

### 3.1. Cotinine and HSA Cys^34^ Adducts by Parent-Reported MSDP

**Figure 1:**
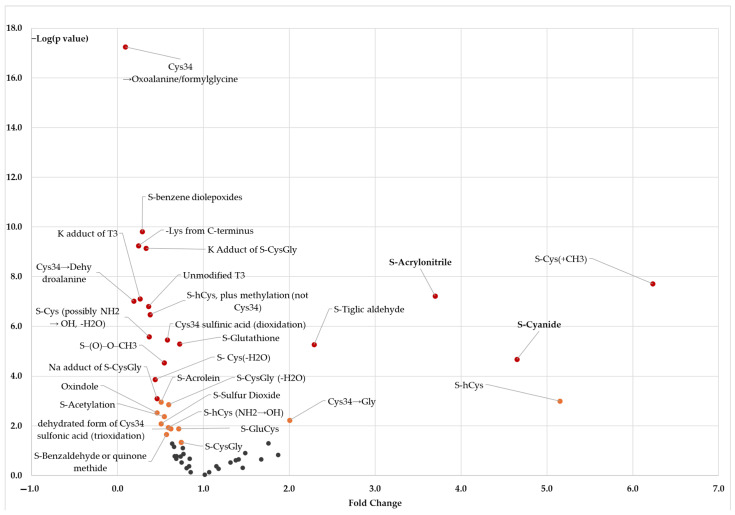
Volcano plot of adducts in newborn blood by presence of any maternal smoking during pregnancy. Orange points represent adducts that were significantly different between newborns unexposed to any MSDP and those exposed at the nominal level (0.001 ≤ *p* < 0.050); Red points represent adducts significant after Bonferroni correction for multiple testing (*p* < 9.1 × 10^−4^).

We compared the levels of Cys^34^ adducts in newborn blood between children ever exposed to MSDP and those who were not (Figure 1). Eighteen adducts exhibited significant differences with MSDP exposure after Bonferroni correction (red points in Figure 1, *p* ≤ 9.1 × 10^−4^). Four (S-Cys(+CH_3_), acrylonitrile, S-tiglic aldehyde, and S-cyanide) showed more than a two-fold increase, and eleven showed more than a 50% decrease between exposed and unexposed groups (red points with fold change < 0.5 in Figure 1). An additional 12 adducts exhibited significant differences with MSDP exposure at the nominal level (orange points in Figure 1, *p* < 0.050). Three adducts varied significantly with trimester-specific MSDP exposure (*p* = 0.007, 0.010, 0.031): the dehydrated form of Cys^34^ sulfinic acid plus methylation (not on Cys^34^), sulfinic acid plus methylation (not Cys^34^), and oxoalanine/formylglycine.

Table 1 presents the level of cotinine and Cys^34^ adducts previously associated with smoking and other environmental exposures, categorized by trimester-specific exposure to MSDP. Cotinine levels were highly elevated in newborns exposed to MSDP during the 3rd trimester (*p* < 0.0001) and slightly elevated among those exposed during the 1st/2nd trimester only (*p* = 0.037), compared to unexposed newborns. Cotinine level also differed significantly between the two exposure groups (*p* < 0.0001). Acrylonitrile levels were similarly elevated in newborns exposed to MSDP during the 3rd trimester (*p* < 0.0001) and those exposed during the 1st/2nd trimester only (*p* = 0.0009). However, acrylonitrile levels did not differ significantly between these two exposure groups (*p* = 0.33). Also, among newborns with 3rd trimester exposure, acrylonitrile level did not vary significantly with the number of cigarettes mothers smoked per day (Figure S1, *p* = 0.16). A similar pattern of association was observed for S-cyanide and S-(N-acetyl) Cys (Table 1).

Ethylene oxide adducts were detected in newborn DBS, but levels did not increase with MSDP exposure during the 3rd trimester (*p* = 0.060). In fact, levels were lower in newborns exposed to MSDP during the 1st/2nd trimester compared to unexposed newborns (*p* = 0.011). Similar pattern was also observed for the adduct at m/z 830.97 (acrolein).

On the other hand, MSDP exposure was associated with lower levels of several adducts, including S-CysGly (-H_2_O), S-Sulfinic acid, and S-γ-GluCys, and dehydrated form of Cys^34^ Sulfonic Acid (*p* = 0.013, <0.0001, 0.0131, and 0.0133, respectively). However, there were no significant differences in these adducts by trimester of MSDP exposure (all Ps > 0.050). Similar patterns, though less statistically significant, were observed for S-crotonaldehyde and S-CysGly.

### 3.2. Acrylonitrile Level in Relationship to Cotinine Level and Parent-Reported MSDP

Among children without parent-reported MSDP, those with MSDP reported for the 1st and 2nd trimester only, and those with MSDP reported for the 3rd trimester, 5% (N = 1), 43% (N = 8), and 83% (N = 38), respectively, had cotinine levels ≥6ng/mL in newborn DBS, a level indicative of active maternal smoking in the week prior to DBS collection (Table 2). Notably, acrylonitrile level was elevated in some children whose mothers reported smoking only during the 1st/2nd trimester and who did not have elevated cotinine level, with some levels approaching those observed among infants with 3rd trimester MSDP and elevated cotinine.

### 3.3. Correlations of HSA Cys^34^ Adducts with Acrylonitrile, Cotinine, Gestational Age, and Parent-Reported MSDP

**Figure 2:**
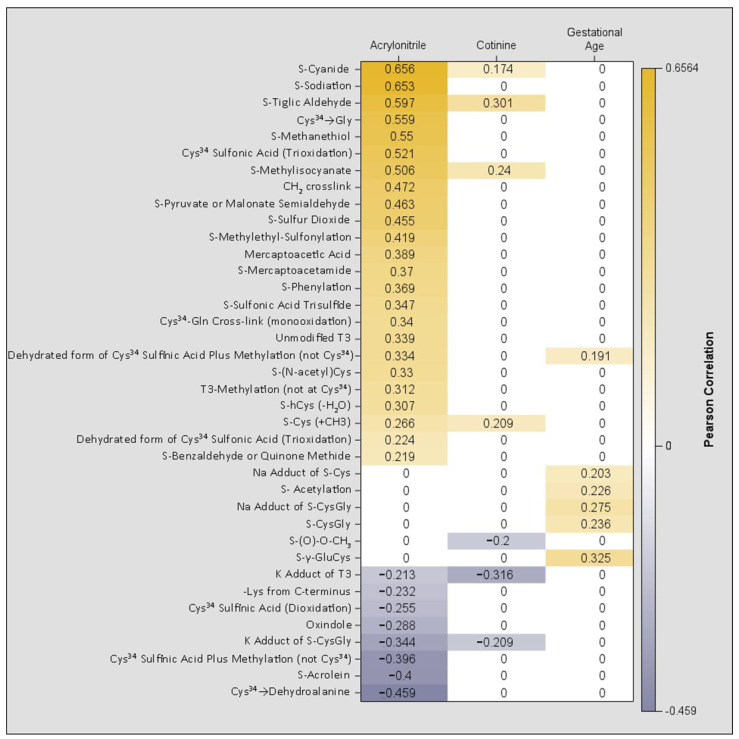
Heatmap of Pearson Correlations Between Cys^34^ Adducts Significantly Associated with Acrylonitrile, Cotinine, and Gestational Age. A value of “0” does not signify a lack of correlation; rather, it indicates that the correlation coefficient did not reach statistical significance (*p* > 0.05). Displayed correlation values are restricted to those with *p* < 0.05.

Given the differing patterns of association between acrylonitrile and cotinine with parent-reported MSDP, we further examined how each relates to other Cys^34^ adducts (Figure 2). For context, associations between Cys^34^ adducts and gestational age are also presented.

Distinct patterns of association were observed between Cys^34^ adducts and acrylonitrile compared to cotinine. Acrylonitrile was positively correlated with 24 adducts (e.g., S-Cyanide, S-(N-acetyl) Cys, sulfonic acid) and negatively correlated with 8 adducts (e.g., sulfinic acid). In contrast, cotinine was positively correlated with only 7 adducts, 6 of which overlapped with those linked to acrylonitrile. Six adducts demonstrated a positive relationship with gestational age, only one of which overlapped with those associated with acrylonitrile and none overlapped with those related to cotinine. Additionally, ethylene oxide showed no association with either cotinine or acrylonitrile.

**Figure 3:**
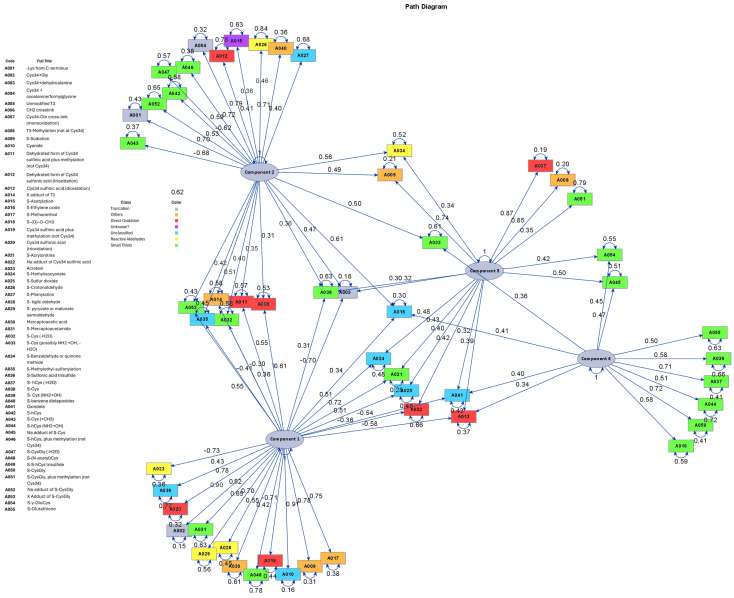
Path Diagram of the Correlation of Cys^34^ Adducts and the Top Four Principal Components.

We performed PC analysis (Table S3) to reduce the dimensionality of Cys^34^ adduct data, excluding acrylonitrile and ethylene oxide. Eight PCs had eigenvalues ≥ 1.0, collectively accounting for 65% of the total variance. Figure 3 shows the loading patterns of Cys^34^ adducts on the top four PCs. PC1 captured variations primarily from direct oxidation products and adducts formed with reactive aldehydes, while PC2 and PC4 were dominated by adducts formed with small thiol compounds. PC1 was strongly and positively associated with acrylonitrile and moderately negatively with ethylene oxide, but was not associated with cotinine and parent-reported MSDP status (Table 3). PC2 showed negative associations with both cotinine level and parent-reported MSDP status, but was not associated with acrylonitrile levels. On the other hand, PC4 was positively associated with acrylonitrile and ethylene oxide but showed no association with cotinine and parent-reported MSDP status.

## 4. Discussion

In this study, we detected a broad range of Cys^34^ adducts in dried blood spots from newborns using advanced mass spectrometry and analytical methods. While some of these chemicals have been studied in blood and urinary samples from adults or adolescents [16], our study contributes essential data on their presence in newborn blood, where the mother serves as a metabolic intermediary for external exposures such as tobacco smoke. Our findings indicate that measurement of acrylonitrile adducts may allow more precise quantification of MSDP exposure, while oxidative stress-related Cys^34^ adducts reflect correlated biological responses. Together, these complementary indicators offer a more comprehensive understanding of the impact of MSDP on fetal metabolic profiles.

Using a targeted, high-sensitivity QqQ method, we detected two constituents of cigarette smoke—ethylene oxide and acrylonitrile—in newborn blood. They were also identified in the cord blood of both preterm and full-term infants in another study that employed similar adductomics technologies [13]. The challenges of detecting these compounds in newborn DBS may help explain why they were not observed in the earlier study by Yano et al. [10] More importantly, we demonstrated that Cys^34^ adducts of acrylonitrile were significantly more abundant in newborns of formerly and currently smoking mothers. These elevated levels of acrylonitrile associated with MSDP are consistent with findings from previous studies in adolescents and adults [8]. We also found that acrylonitrile levels were elevated in some newborns of former smoking mothers, indicating possible exposure to environmental smoke from secondary (other household smokers) or tertiary (contaminated household furnishings) sources. Because of the addictiveness of nicotine, the self-report of quitting smoking among our former smokers could be doubted, but the low cotinine levels in the blood of these former smoking mothers compared to current smokers support a lack of active smoking in these subjects. Unfortunately, information regarding household smoking by individuals other than mothers was not available in our study. Consequently, we could not account for the potential confounding effects of total environmental tobacco smoke exposure.

Conversely, we found no association between ethylene oxide and parent-reported MSDP exposure, a finding that contrasts with studies in older populations. Elevated levels of ethylene oxide adducts have been found in the blood of smokers [8], including both adolescents and adults [17]. One possible explanation is that the placenta may act as a barrier, filtering out elevated ethylene oxide present in smoking mothers. The lack of association between ethylene oxide and acrylonitrile adducts, coupled with its association with other Cys^34^ adducts in newborn blood, suggests that the ethylene oxide adducts detected in newborns may instead result from endogenous production, potentially as a byproduct of ethylene metabolism.

Consistent with Yano et al. [10], we found that the putative acrolein adduct (m/z = 830.97) was lower among newborns with parent-reported MSDP. Furthermore, we found that the putative acrolein adduct was negatively associated with acrylonitrile levels and not associated with cotinine level. However, acrolein, a known component of cigarette smoke, has been found at elevated levels in the urine of smokers [12,16,18]. These discrepancies highlight the need for further research to better understand the behavior of acrolein-related adducts in the fetal environment and their relationship to prenatal exposure to tobacco smoke.

The Cys^34^ adduct of cyanide, which results from exposure to maternal inhalation of hydrogen cyanide in tobacco smoke, has been shown to be a reliable biomarker to discriminate between newborns of smoking and nonsmoking mothers [10]. Consistent with earlier findings, we also observed elevated Cys^34^ adducts of cyanide in newborns with parent-reported MSDP. Furthermore, S-cyanide levels were strongly associated with acrylonitrile levels and only mildly associated with cotinine levels, suggesting that this adduct may reflect chronic rather than recent exposure to tobacco smoke during pregnancy.

The adducts detected in newborn DBS also reflected ROS responses correlated with MSDP, consistent with previous studies. Specifically, we observed reduced levels of S-Sulfinic acid, and S-γGluCys, and elevated levels of S-(N-acetyl) Cys in newborns with MSDP exposure. These observations align with the findings of Grigoryan et al. [8], who reported reduced S-Sulfinic acid and elevated S-(N-acetyl) Cys in adult smokers, as well as with a previous report linking reduced levels S-sulfinic acid and S-γ-GluCys in association with prenatal air pollution exposure [9]. Interestingly, our data further demonstrated that S-Sulfinic acid levels were negatively associated and sulfonic acid and S-(N-acetyl) Cys levels positively associated with acrylonitrile levels, but were not associated with gestational age. In contrast, S-γ-GluCys was not associated with acrylonitrile but was positively associated with gestational age. These findings suggest that alterations in S-Sulfinic acid, sulfonic acid, and S-(N-acetyl) Cys may reflect biological responses to chronic MSDP exposure, whereas changes in S-γ-GluCys may be influenced by other MSDP-related factors, such as preterm birth. Notably, we identified five additional adducts, including S-CysGly and S-acetylation, that were also significantly associated with gestational age. This suggests that these specific adducts may serve as biomarkers for the metabolic maturity of newborns. However, further research is needed to better understand the dynamic relationships among the various Cys^34^ adducts. Our analyses revealed distinct underlying structures in the adduct profile, suggesting coordinated biological responses.

In assessing biological responses to MSDP, acrylonitrile adduct levels may serve as a more comprehensive and supportable indicator than cotinine levels. In our study, acrylonitrile exhibited a broader range of significant associations with Cys^34^ adducts compared to cotinine. Notably, the PCs of Cys^34^ adducts associated with cotinine were also associated with acrylonitrile and parent-reported MSDP. In other words, acrylonitrile, in conjunction with parent-reported MSDP, was associated with the top four PCs of the Cys^34^ adduct profile, which collectively accounted for over 50% of the total variance. This difference between acrylonitrile and cotinine may reflect the longer biological half-life of acrylonitrile, allowing it to capture cumulative in utero exposure more effectively, whereas cotinine, a short-lived metabolite, primarily reflects more recent exposure.

Our findings may also inform public health policies and future research on the impact of emerging tobacco products. Although e-cigarettes are often marketed as safer alternatives to combustible tobacco, they still lead to exposure to harmful compounds such as acrylonitrile [11]. A study of urinary metabolites in adolescent users found that while poly-users (those using both e-cigarettes and combustible cigarettes) had the highest levels of toxic volatile compounds, exclusive e-cigarette users also showed significantly elevated concentrations [19]. For example, metabolites of acrylonitrile, acrolein, and crotonaldehyde were found to be 341%, 20%, and 20% higher, respectively, compared to nonsmoking controls. Notably, fruit-flavored e-cigarettes—which are popular among teens—were associated with even greater acrylonitrile levels [11]. As this demographic ages, the potential for fetal exposure via maternal e-cigarette use may increase [20].

This study has several strengths, including measurement of a large panel of adducts in newborn blood and the ability to compare these biomarkers with both self-reported maternal smoking during pregnancy and cotinine levels in newborn blood. However, several limitations should be considered. First, the analysis was based on a relatively small sample (n = 110), drawn from a convenience sample of children with and without ocular diseases. In addition, the majority of participants were African American. These factors may limit generalizability and statistical power. In addition, although we adjusted for gestational age when evaluating associations between adducts and maternal smoking, we could not account for other potential confounders, including secondhand smoke exposure, maternal diet, and ambient air pollution, all of which may influence adduct levels.

## 5. Conclusions

Our study provides new insights into Cys^34^ adducts in newborn blood and their associations with parent-reported MSDP and cotinine levels. The findings underscore the potential of Cys^34^ adducts as biomarkers of both environmental exposures—such as MSDP—and related biological responses, supporting their utility in developmental research. Further studies in larger and more diverse populations are warranted to validate these findings, deepen our understanding of the temporal dynamics of adduct accumulation in the developing fetus, assess risks associated with emerging exposures such as e-cigarette use during pregnancy, and broaden the applications of newborn blood analysis in early-life exposure assessment.

## Figures and Tables

**Table 1:** Levels of Cotinine and Cys^34^ Adducts in Newborn Blood by Parent-reported Maternal Smoking During Different Trimesters of Pregnancy (MSDP).

	Geometric Mean (Standard Deviation) ^1^		
	No MSDP (N = 20)	1st/2nd Trimester MSDP (N = 44)	3rd Trimester MSDP (N = 46)
Cotinine, ng/mL	3.14 (3.18)	6.92 (4.33) *^2^	29.07 (4.07) **^2^
**Cys^34^ adducts, PAR:**			
*S*-Acrylonitrile	0.209 (1.98)	0.675 (4.05) **	0.878 (3.85) **
*S*-Ethylene oxide	1.036 (2.01)	0.548 (2.63) *	0.789 (2.56)
*S*-Cyanide	0.129 (3.01)	0.652 (8.88) *	0.552 (7.41) *
*S*-Acrolein	0.547 (1.88)	0.257 (4.44) *	0.298 (3.23)
*S*-Crotonaldehyde	3.082 (3.11)	2.207 (2.10)	2.032 (1.90)
*S*-Sulfinic acid	21.59 (1.40)	11.59 (2.35) **	13.56 (1.73) *
*S*-GluCys	0.496 (1.85)	0.358 (1.84) *	0.350 (1.54) *
Dehydrated form of *S*-Sulfonic Acid	0.596 (2.05)	0.322 (1.97) *	0.426 (2.30)
*S*-(*N*-acetyl)Cys	0.045 (6.15)	0.081 (2.37) *	0.087 (2.04) *
*S*-CysGly-(H_2_O)	0.167 (1.89)	0.097 (1.89) *	0.103 (1.89) *
*S*-CysGly	2.639 (1.93)	1.934 (1.76)	1.985 (1.80)

^1^ Levels of Cys^34^ adducts: Peak area ratio (PAR) ×1000. Geometric mean and standard deviation were estimated from natural log-transformed data and then exponentially transformed for table presentation. ^2^ P for difference compared with no MSDP was determined by *t*-test of log-transformed values. *: 0.001 ≤ *p* < 0.050; **: *p* < 0.001.

**Table 2:** Levels of Acrylonitrile Adduct in Newborn Blood among Children with Different Parent-reported Maternal Smoking During Pregnancy and Cotinine Level.

Parent-Reported MSDP	Cotinine Level	N (%)	Acrylonitrile Adduct Level ^1^		
			Mean ± SD	Range	*p*
No	<6 ng/mL	19 (95%)	0.203 ± 1.995	0.070–1.192	Reference
	≥6 ng/mL	1 (5%)	0.361 ± 0	0.361–0.361	-
1st/2nd Trimester smoking only	<6 ng/mL	25 (57%)	0.463 ± 4.184	0.047–5.730	0.44
	≥6 ng/mL	19 (43%)	1.110 ± 3.375	0.048–10.606	0.019
3rd trimester smoking	<6 ng/mL	8 (17%)	0.573 ± 7.412	0.034–7.362	0.071
	≥6 ng/mL	38 (83%)	0.960 ± 3.270	0.033–7.982	0.022

^1^ Peak area ratio (PAR) ×1000. Geometric mean and standard deviation (SD) were estimated from natural log-transformed data and then exponentially transformed for table presentation.

**Table 3:** Association of Top Principal Component Analysis Factors with Different Measures/biomarkers of Maternal Smoking During Pregnancy.

	PC1	PC2	PC3	PC4
Cotinine, ng/mL				
Correlation Coefficient ^1^	0.124	−0.253	0.202	0.013
*p*-Value ^1^	0.19	0.0076	0.034	0.89
S-Acrylonitrile				
Correlation Coefficient ^1^	0.662	−0.164	0.372	0.289
*p*-Value ^1^	<0.0001	0.086	<0.0001	0.0022
Parent-reported MSDP: yes vs. no				
Regression Coefficient ^2^	0.380	−1.659	0.123	−0.183
*p*-Value ^1^	0.1244	<0.0001	0.62	0.46
Parent-reported MSDP: 3rd Trimester vs. no				
Regression Coefficient ^2^	0.123	−0.883	0.133	−0.059
*p*-Value ^1^	0.30	<0.0001	0.284	0.65
Ethylene Oxide				
Correlation Coefficient ^1^	−0.350	−0.030	0.315	0.314
*p*-Value ^1^	0.0002	0.76	0.0008	0.0008

Abbreviations: MSDP = maternal smoking during pregnancy; PC = principal Component. ^1^ Pearson correlation coefficient and corresponding *p* values. ^2^ Regression coefficient and *p* values from logistic regression models of smoking outcomes on the PCs.

## Data Availability

Restrictions apply to the availability of these data. Newborn dried blood spots were obtained from the California’s Biobank Program and are available from the authors with the permission of the California’s Biobank Program and the Committee for the Protection of Human Subjects of the State of California.

## Supplementary Materials

The following supporting information can be downloaded at: https://www.mdpi.com/article/10.3390/antiox15040411/s1, Figure S1: Levels of Acrylonitrile Adduct (Peak-to-area ratio, log-transformed) and Cotinine (ng/mL) in newborn blood by the timing and amount of parent-reported maternal smoking during pregnancy; Table S1: Detailed T3 peptides detected in samples of smokers and non-smokers; Table S2: Demographic Characteristics of Study Cohort by Parent-reported Maternal Smoking during Pregnancy. Table S3: Principal Component Analysis of 53 DBS HSA-Cys^34^ Adducts.

## Abbreviations

The following abbreviations are used in this manuscript:

MSDP	Maternal smoking during pregnancy
DBS	Dried blood spots
HSA	Human serum albumin
QqQ	Triple-quadrupole
MS	Mass spectrometry
MEPEDS	The Multiethnic Pediatric Eye Disease Study
GDSP	Genetic Disease Screening Program
MRM	Multiple Reaction Monitoring
PAR	Peak area ratio
PC	Principal component
