# Supplementary material for: Adductomics of Newborn Dried Blood Spots Detects Constituents of Maternal Smoking During Pregnancy and Associated Oxidative Stress Exposure
Source: Antioxidants (Basel). 2026 Mar 25;15(4):411. doi: 10.3390/antiox15040411 (PMC13113834; doi:10.3390/antiox15040411)
Supplement: Supplementary file 1 [file antioxidants-15-00411-s001.zip › antioxidants-4191127-supplementary.pdf]

Supplemental Tables and Figures:

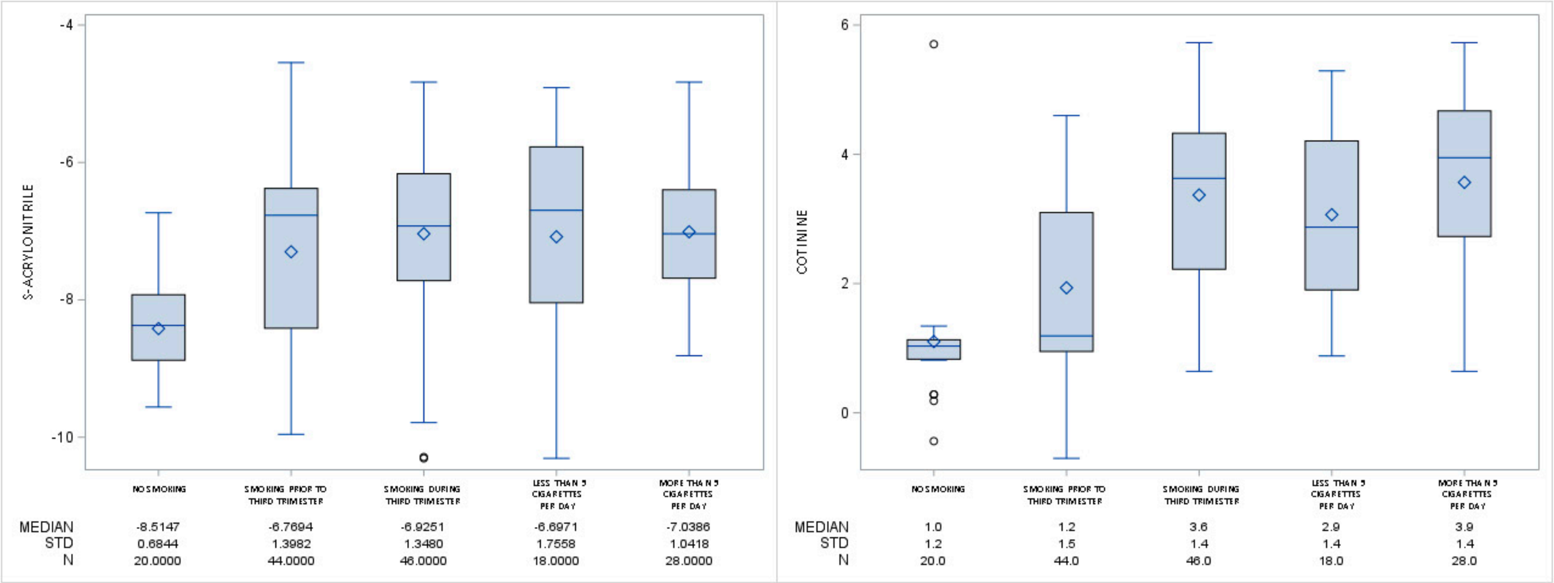

Figure S1. Levels of Acrylonitrile Adduct (Peak to area ratio, log transformed) and Cotinine (ng/ml) in newborn blood by the timing and amount of parent-reported maternal smoking during pregnancy

Table S1. Detailed T3 peptides detected in samples of smokers and non-smokers

Label	Putative Annotation	m/z	Added Mass (Da)	QQQ retention Time (min)	PAR (x 1,000) overall, median (range)
A001	-Lys from C-terminus	769.59	-128.10	6.70	1.474 (0.101, 18.346)
A002	Cys^{34}→Gly	796.96	-45.99	4.75	0.147 (0.007, 99.586)
A003	Cys^{34}→dehydroalanine	800.96	-33.99	5.20	0.891 (0.020, 15.611)
A004	Cys^{34} → oxoalanine/formylglycine	806.29	-18.00	5.10	1.737 (0.156, 233.961)
A005	Unmodified T3	812.29	1.01	5.16	246.327 (7.077, 1003.9)
A006	CH_2 crosslink	815.95	12.00	4.70	0.219 (0.020, 2.860)
A007	Cys^{34}-Gln cross-link (monooxidation)	816.65	13.98	5.35	11.669 (0.263, 58.852)
A008	T3-Methylation (not at Cys^{34})	816.96	15.02	5.32	9.953 (0.071, 56.827)
A009	*S*-Sodiation	819.62	22.99	5.16	0.985 (0.0001, 54.979)
A010	S-Cyanide	820.62	26.00	5.15	0.233 (0.007, 21.283)
A011	Dehydrated form of Cys^{34} sulfinic acid plus methylation (not Cys^{34})	821.96	28.99	3.50	0.215 (0.018, 4.236)
A012	Dehydrated form of Cys^{34} sulfonic acid (trioxidation)	822.62	30.97	4.55	233.8 (0.057, 6.336)
A013	Cys^{34} sulfinic acid (dioxidation)	822.95	32.99	4.42	15.651 (0.098, 32.941)
A014	K adduct of T3	824.98	38.96	5.16	0.085 (0.0002, 2.204)
A015	S-Acetylation	826.30	43.02	5.20	0.353 (0.019, 3.013)
A016	S-Ethylene oxide	826.97	45.03	4.96	0.774 (0.050, 3.397)
A017	S-Methanethiol	827.62	47.00	4.99	0.209 (0.005, 1.886)
A018	S–(O)–O–CH_3	827.63	47.01	4.50	0.954 (0.321, 4.731)
A019	Cys^{34} sulfinic acid plus methylation (not Cys^{34})	827.96	47.00	4.40	1.585 (0.094, 6.100)
A020	Cys^{34} sulfonic acid (trioxidation)	828.29	48.99	4.49	0.741 (0.062, 3.981)
A021	S-Acrylonitrile	829.97	54.04	5.10	0.778 (0.033, 10.606)
A022	Na adduct of Cys^{34} sulfinic acid	830.62	54.98	4.50	0.926 (0.144, 4.844)
A023	Acrolein	830.97	57.03	4.50	0.362 (0.020, 3.648)
A024	S-Methylisocyanate	831.30	58.03	5.15	0.235 (0.0002, 2.326)
A025	S-Sulfur dioxide	833.64	64.97	5.10	0.091 (0.0001, 0.963)
A026	S-Crotonaldehyde	835.65	71.05	5.28	2.241 (0.175, 26.352)
A027	S-Phenylation	837.65	77.04	5.10	0.159 (0.018, 1.783)
A028	S- tiglic aldehyde	840.33	85.06	4.35	0.306 (0.035, 2.591)
A029	S- pyruvate or malonate semialdehyde	841.64	89.02	4.90	0.086 (0.0001, 117.762)

Label	Putative Annotation	m/z	Added Mass (Da)	QQQ retention Time (min)	PAR (x 1,000) overall, median (range)
A030	Mercaptoacetic acid	842.33	90.00	4.88	0.13 (0.003, 3.808)
A031	S-Mercaptoacetamide	842.32	90.98	4.95	0.201 (0.002, 7.408)
A032	S-Cys (-H_2O)	846.00	102.00	4.15	0.633 (0.131, 7.300)
A033	S-Cys (possibly NH_2 →OH, -H_2O)	846.66	102.98	5.50	0.24 (0.00005, 1.540)
A034	S-Benzaldehyde or quinone methide	847.66	107.01	3.40	0.37 (0.038, 3.887)
A035	S-Methylethyl-sulfonylation	848.00	107.05	5.50	0.117 (0.0001, 1.507)
A036	S-Sulfonic acid trisulfide	849.66	112.94	4.50	0.095 (0.0001, 0.382)
A037	S- hCys (-H_2O)	850.67	116.02	4.30	0.285 (0.008, 1.929)
A038	S-Cys	852.00	120.01	3.57	8.156 (2.719, 32.12)
A039	S- Cys (NH_2→OH)	852.67	121.00	3.60	2.409 (0.0001, 9.277)
A040	S-benzene diolepoxides	855.00	129.05	3.20	0.141 (0.008, 1.137)
A041	Oxindole	856.00	132.04	3.80	0.359 (0.015, 2.681)
A042	S-hCys	856.68	134.02	3.62	0.243 (0.0002, 5.560)
A043	S-Cys (+CH3)	856.68	134.02	3.65	0.609 (0.006,6.302)
A044	S-hCys (NH_2→OH)	857.34	135.01	4.25	2.24 (0.147, 7.533)
A045	Na adduct of S-Cys	859.33	141.99	3.57	1.045 (0.088, 4.573)
A046	S-hCys, plus methylation (not Cys[34])	861.35	148.03	3.85	0.115 (0.016, 0.706)
A047	S-CysGly (-H_2O)	865.01	159.02	3.75	0.108 (0.012, 0.495)
A048	S-(N-acetyl)Cys	866.01	162.02	4.30	0.074 (0.0002, 0.581)
A049	S-S-hCys trisulfide	867.36	166.00	3.70	0.076 (0.0001, 0.517)
A050	S-CysGly	871.02	177.03	3.30	2.068 (0.558, 10.544)
A051	S-CysGly, plus methylation (not Cys[34])	875.69	191.04	3.40	0.339 (0.031, 2.427)
A052	Na adduct of S-CysGly	878.68	199.01	3.40	0.174 (0.0147, 4.313)
A053	K Adduct of S-CysGly	884.05	214.98	3.40	0.087 (0.012, 0.618)
A054	S-γ-GluCys	895.04	249.05	3.68	0.387 (0.044, 1.681)
A055	S-Glutathione	914.06	306.07	3.54	33.18 (11.37, 61.05)

Table S2. Demographic Characteristics of Study Cohort by Parent-reported Maternal Smoking during Pregnancy.

	N (column percent)	N (row percent)			
	**Total** (N=110)	**No maternal smoking during pregnancy** (N=20)	**Maternal Smoking only in 1st/2nd Trimester** (N=44)	**Maternal Smoking in all three Trimesters** (N=46)	**P for difference**[1]
**Sex**					
Male	62 (56.4%)	8 (12.9%)	28 (45.2%)	26 (41.9%)	0.18
Female	48 (43.6%)	12 (25.0%)	16 (33.3%)	20 (41.7%)	
**Race/ethnicity groups**					
Non-Hispanic White	14 (12.7%)	5 (35.7%)	7 (50.0%)	2 (14.3%)	<0.001
African American	94 (85.5%)	14 (14.9%)	36 (38.3%)	44 (46.8%)	
Hispanic White	2 (1.8%)	1 (50.0%)	1 (50.0%)	-	
**Gestational age**					
> 37 Weeks	90 (81.8%)	19 (21.1%)	36 (40.0%)	35 (38.9%)	<0.001
37 to 32 Weeks	14 (12.7%)	1 (7.1%)	6 (42.9%)	7 (50.0%)	
< 32 Weeks	6 (5.5%)	-	2 (33.3%)	4 (66.7%)	

[1]P for difference was estimated from Chi-squared test.

Table S3. Principal Component Analysis of 53 DBS HSA-Cys^{34}Adducts

	Eigen value	Difference	Proportion	Cumulative
Factor 1	11.6	2.7	0.21	0.21
Factor 2	8.9	4.2	0.16	0.37
Factor 3	4.7	1.9	0.09	0.46
Factor 4	2.8	0.4	0.05	0.51
Factor 5	2.4	0.04	0.04	0.55
Factor 6	2.4	0.9	0.04	0.60
Factor 7	1.5	0.2	0.03	0.62
Factor 8	1.3	0.09	0.02	0.65
